# Synaptic processes and immune-related pathways implicated in Tourette syndrome

**DOI:** 10.1038/s41398-020-01082-z

**Published:** 2021-01-18

**Authors:** Fotis Tsetsos, Dongmei Yu, Jae Hoon Sul, Alden Y. Huang, Cornelia Illmann, Lisa Osiecki, Sabrina M. Darrow, Matthew E. Hirschtritt, Erica Greenberg, Kirsten R. Muller-Vahl, Manfred Stuhrmann, Yves Dion, Guy A. Rouleau, Harald Aschauer, Mara Stamenkovic, Monika Schlögelhofer, Paul Sandor, Cathy L. Barr, Marco A. Grados, Harvey S. Singer, Markus M. Nöthen, Johannes Hebebrand, Anke Hinney, Robert A. King, Thomas V. Fernandez, Csaba Barta, Zsanett Tarnok, Peter Nagy, Christel Depienne, Yulia Worbe, Andreas Hartmann, Cathy L. Budman, Renata Rizzo, Gholson J. Lyon, William M. McMahon, James R. Batterson, Danielle C. Cath, Irene A. Malaty, Michael S. Okun, Cheston Berlin, Douglas W. Woods, Paul C. Lee, Joseph Jankovic, Mary M. Robertson, Donald L. Gilbert, Lawrence W. Brown, Barbara J. Coffey, Andrea Dietrich, Pieter J. Hoekstra, Samuel Kuperman, Samuel H. Zinner, Michael Wagner, James A. Knowles, A. Jeremy Willsey, Jay A. Tischfield, Gary A. Heiman, Nancy J. Cox, Nelson B. Freimer, Benjamin M. Neale, Lea K. Davis, Giovanni Coppola, Carol A. Mathews, Jeremiah M. Scharf, Peristera Paschou, on behalf of the Tourette Association of America International Consortium for Genetics, Cathy L. Barr, James R. Batterson, Cheston Berlin, Cathy L. Budman, Danielle C. Cath, Giovanni Coppola, Nancy J. Cox, Sabrina Darrow, Lea K. Davis, Yves Dion, Nelson B. Freimer, Marco A. Grados, Erica Greenberg, Matthew E. Hirschtritt, Alden Y. Huang, Cornelia Illmann, Robert A. King, Roger Kurlan, James F. Leckman, Gholson J. Lyon, Irene A. Malaty, Carol A. Mathews, William M. McMahon, Benjamin M. Neale, Michael S. Okun, Lisa Osiecki, Mary M. Robertson, Guy A. Rouleau, Paul Sandor, Jeremiah M. Scharf, Harvey S. Singer, Jan H. Smit, Jae Hoon Sul, Dongmei Yu, the Gilles de la Tourette GWAS Replication Initiative, Harald Aschauer Harald Aschauer, Csaba Barta, Cathy L. Budman, Danielle C. Cath, Christel Depienne, Andreas Hartmann, Johannes Hebebrand, Anastasios Konstantinidis, Carol A. Mathews, Kirsten Müller-Vahl, Peter Nagy, Markus M. Nöthen, Peristera Paschou, Renata Rizzo, Guy A. Rouleau, Paul Sandor, Jeremiah M. Scharf, Monika Schlögelhofer, Mara Stamenkovic, Manfred Stuhrmann, Fotis Tsetsos, Zsanett Tarnok, Tomasz Wolanczyk, Yulia Worbe, the Tourette International Collaborative Genetics Study, Lawrence Brown, Keun-Ah Cheon, Barbara J. Coffey, Andrea Dietrich, Thomas V. Fernandez, Blanca Garcia-Delgar, Donald Gilbert, Dorothy E. Grice, Julie Hagstrøm, Tammy Hedderly, Gary A. Heiman, Isobel Heyman, Pieter J. Hoekstra, Chaim Huyser, Young Key Kim, Young-Shin Kim, Robert A. King, Yun-Joo Koh, Sodahm Kook, Samuel Kuperman, Bennett L. Leventhal, Marcos Madruga-Garrido, Pablo Mir, Astrid Morer, Alexander Münchau, Kerstin J. Plessen, Veit Roessner, Eun-Young Shin, Dong-Ho Song, Jungeun Song, Jay A. Tischfield, A. Jeremy Willsey, Samuel Zinner, and the Psychiatric Genomics Consortium Tourette Syndrome Working Group, Harald Aschauer, Cathy L. Barr, Csaba Barta, James R. Batterson, Cheston Berlin, Lawrence Brown, Cathy L. Budman, Danielle C. Cath, Barbara J. Coffey, Giovanni Coppola, Nancy J. Cox, Sabrina Darrow, Lea K. Davis, Christel Depienne, Andrea Dietrich, Yves Dion, Thomas Fernandez, Nelson B. Freimer, Donald Gilbert, Marco A. Grados, Erica Greenberg, Andreas Hartmann, Johannes Hebebrand, Gary Heiman, Matthew E. Hirschtritt, Pieter Hoekstra, Alden Y. Huang, Cornelia Illmann, Joseph Jankovic, Robert A. King, Samuel Kuperman, Paul C. Lee, Gholson J. Lyon, Irene A. Malaty, Carol A. Mathews, William M. McMahon, Kirsten Müller-Vahl, Peter Nagy, Benjamin M. Neale, Markus M. Nöthen, Michael S. Okun, Lisa Osiecki, Peristera Paschou, Renata Rizzo, Mary M. Robertson, Guy A. Rouleau, Paul Sandor, Jeremiah M. Scharf, Monika Schlögelhofer, Harvey S. Singer, Mara Stamenkovic, Manfred Stuhrmann, Jae Hoon Sul, Zsanett Tarnok, Jay Tischfield, Fotis Tsetsos, A. Jeremy Willsey, Douglas Woods, Yulia Worbe, Dongmei Yu, Samuel Zinner

**Affiliations:** 1 Department of Molecular Biology and Genetics, Democritus University of Thrace, Alexandroupolis, Greece; 2 Psychiatric and Neurodevelopmental Genetics Unit, Center for Genomic Medicine, Department of Psychiatry, Massachusetts General Hospital, Boston, MA USA; 3 Stanley Center for Psychiatric Research, Broad Institute of MIT and Harvard, Cambridge, MA USA; 4 Semel Institute for Neuroscience and Human Behavior, David Geffen School of Medicine, University of California, Los Angeles, CA USA; 5 Department of Psychiatry and Biobehavioral Sciences, University of California, Los Angeles, CA USA; 6 Bioinformatics Interdepartmental Program, University of California, Los Angeles, CA USA; 7 Department of Psychiatry, UCSF Weill Institute for Neurosciences, University of California, San Francisco, CA USA; 8 Department of Psychiatry, Massachusetts General Hospital, Boston, MA USA; 9 Clinic of Psychiatry, Social Psychiatry, and Psychotherapy, Hannover Medical School, Hannover, Germany; 10 Institute of Human Genetics, Hannover Medical School, Hannover, Germany; 11 McGill University Health Center, University of Montreal, McGill University Health Centre, Montreal, Canada; 12 Montreal Neurological Institute, Department of Neurology and Neurosurgery, McGill University, Montreal, Canada; 13 Department of Psychiatry and Psychotherapy, Medical University Vienna, Vienna, Austria; 14 Biopsychosocial Corporation, Vienna, Austria; 15 University Health Network, Youthdale Treatment Centres, and University of Toronto, Toronto, Canada; 16 Krembil Research Institute, University Health Network, Hospital for Sick Children, and University of Toronto, Toronto, Canada; 17 Johns Hopkins University School of Medicine and the Kennedy Krieger Institute, Baltimore, MD USA; 18 Institute of Human Genetics, University Hospital Bonn, University of Bonn Medical School, Bonn, Germany; 19 Department of Child and Adolescent Psychiatry, Psychosomatics, and Psychotherapy, University Hospital Essen, University of Duisburg-Essen, Essen, Germany; 20 Yale Child Study Center and the Department of Psychiatry, Yale University School of Medicine, New Haven, CT USA; 21 Institute of Medical Chemistry, Molecular Biology, and Pathobiochemistry, Semmelweis University, Budapest, Hungary; 22 Vadaskert Child and Adolescent Psychiatric Hospital, Budapest, Hungary; 23 Institute of Human Genetics, University Hospital Essen, University Duisburg-Essen, Essen, Germany; 24 Sorbonne Universités, UPMC Université Paris 06, UMR S 1127, CNRS UMR 7225 ICM Paris, France; 25 French Reference Centre for Gilles de la Tourette Syndrome, Groupe Hospitalier Pitié-Salpêtrière, Paris, France; 26 Assistance Publique–Hôpitaux de Paris, Department of Neurology, Groupe Hospitalier Pitié-Salpêtrière, Paris, France; 27 Assistance Publique Hôpitaux de Paris, Hopital Saint Antoine, Paris, France; 28 Zucker School of Medicine at Hofstra/Northwell, Hempstead, NY USA; 29 Child Neuropsychiatry, Department of Clinical and Experimental Medicine, University of Catania, Catania, Italy; 30 Jervis Clinic, NYS Institute for Basic Research in Developmental Disabilities (IBR), Staten Island, NY USA; 31 Department of Psychiatry, University of Utah, Salt Lake City, UT USA; 32 Children’s Mercy Hospital, Kansas City, MO USA; 33 Department of Psychiatry, University Medical Center Groningen and Rijksuniversity Groningen, and Drenthe Mental Health Center, Groningen, the Netherlands; 34 Department of Neurology, Norman Fixel Institute for Neurological Diseases, University of Florida Health, Gainesville, FL USA; 35 Pennsylvania State University College of Medicine, Hershey, PA USA; 36 Marquette University and University of Wisconsin-Milwaukee, Milwaukee, WI USA; 37 Tripler Army Medical Center and University of Hawaii John A. Burns School of Medicine, Honolulu, HI USA; 38 Parkinson’s Disease Center and Movement Disorders Clinic, Department of Neurology, Baylor College of Medicine, Houston, TX USA; 39 Division of Psychiatry, Department of Neuropsychiatry, University College London, London, UK; 40 Division of Pediatric Neurology, Cincinnati Children’s Hospital Medical Center; Department of Pediatrics, University of Cincinnati, Cincinnati, USA; 41 Children’s Hospital of Philadelphia, Philadelphia, PA USA; 42 Department of Psychiatry and Behavioral Sciences, University of Miami Miller School of Medicine, Miami, FL USA; 43 Department of Child and Adolescent Psychiatry, University Medical Center Groningen, University of Groningen, Groningen, the Netherlands; 44 University of Iowa Carver College of Medicine, Iowa City, IA USA; 45 Department of Pediatrics, University of Washington, Seattle, WA USA; 46 Department of Psychiatry and Psychotherapy, University Hospital Bonn, Bonn, Germany; 47 SUNY Downstate Medical Center Brooklyn, Brooklyn, NY USA; 48 Institute for Neurodegenerative Diseases, UCSF Weill Institute for Neurosciences, University of California San Francisco, San Francisco, CA USA; 49 Department of Genetics and the Human Genetics Institute of New Jersey, Rutgers, the State University of New Jersey, Piscataway, NJ USA; 50 Division of Genetic Medicine, Vanderbilt Genetics Institute, Vanderbilt University Medical Center, Nashville, TN USA; 51 Analytic and Translational Genetics Unit, Department of Medicine, Massachusetts General Hospital, Boston, MA USA; 52 Department of Psychiatry, Genetics Institute, University of Florida, Gainesville, FL USA; 53 Department of Neurology, Brigham and Women’s Hospital, and the Department of Neurology, Massachusetts General Hospital, Boston, MA USA; 54 Department of Biological Sciences, Purdue University, West Lafayette, IN USA; 55 Atlantic Neuroscience Institute, Overlook Hospital, Summit, NJ USA; 56 Yale Child Study Center, Yale University School of Medicine, New Haven, CT USA; 57 Department of Psychiatry, VU UniversityMedical Center, Amsterdam, The Netherlands; 58 Center for Mental Health Muldenstrasse, BBRZMed, Linz, Austria; 59 Department of Child Psychiatry, Medical University of Warsaw, 00-001 Warsaw, Poland; 60 Assistance Publique Hôpitaux de Paris, Hopital Saint Antoine, Paris, France; 61 Yonsei University College of Medicine, Yonsei Yoo & Kim Mental Health Clinic, Seoul, South Korea; 62 Department of Child and Adolescent Psychiatry and Psychology, Institute of Neurosciences, Hospital Clinic Universitari, Barcelona, Spain; 63 Department of Psychiatry, Friedman Brain Institute, Mindich Child Health and Development Institute, Icahn School of Medicine at Mount Sinai, New York, NY USA; 64 Child and Adolescent Mental Health Center, Mental Health Services, Capital Region of Denmark and University of Copenhagen, Copenhagen, Denmark; 65 Tic and Neurodevelopmental Movements Service (TANDeM), Evelina Children’s Hospital, Guys and St Thomas’ NHS Foundation Trust, London, UK; 66 Paediatric Neurosciences, Kings College London, London, UK; 67 UCL Great Ormond Street Institute of Child Health, University College London, London, UK; 68 Psychological and Mental Health Services, Great Ormond Street Hospital for Children NHS Foundation Trust, London, UK; 69 De Bascule, Academic Centre for Child and Adolescent Psychiatry, Amsterdam, The Netherlands; 70 Yonsei Bom Clinic, Seoul, South Korea; 71 Department of Psychiatry, University of California, San Francisco, San Francisco, CA USA; 72 The Korea Institute for Children’s Social Development, Rudolph Child Research Center, Seoul, South Korea; 73 Kangbuk Samsung Hospital, Seoul, South Korea; 74 Department of Psychiatry, University of California, San Francisco, San Francisco, CA USA; 75 Sección de Neuropediatría, Instituto de Biomedicina de Sevilla, Hospital Universitario Virgen del Rocío/CSIC/Universidad de Sevilla, Seville, Spain; 76 Hospital Universitario Virgen del Rocío, Sevilla, Spain; 77 Centro de Investigación en Red-Enfermedades Neurodegenerativas (CIBERNED), Madrid, Spain; 78 Department of Child and Adolescent Psychiatry and Psychology, Institute of Neurosciences, Hospital Clínic Universitari, Barcelona, Spain; 79 Department of Medicine, University of Barcelona, Barcelona, Spain; 80 Centro de Investigación Biomédica en red de Salud Mental (CIBERSAM), Barcelona, Spain; 81 Institute of Systems Motor Science, University of Lübeck, Lübeck, Germany; 82 Child and Adolescent Mental Health Centre, Mental Health Services, Capital Region of Denmark, Copenhagen, Denmark; 83 The Lundbeck Foundation Initiative for Integrative Psychiatric Research, Aarhus, Denmark; 84 Service of Child and Adolescent Psychiatry, Department of Psychiatry, University Medical Center, University of Lausanne, Lausanne, Switzerland; 85 Department of Child and Adolescent Psychiatry, Faculty of Medicine, University Hospital Carl Gustav CarusTU Dresden, Dresden, Germany; 86 Yonsei University College of Medicine, Yonsei Yoo & Kim Mental Health Clinic, Seoul, South Korea; 87 Yonsei University College of Medicine, Yonsei Yoo & Kim Mental Health Clinic, Seoul, South Korea; 88 National Health Insurance Service Ilsan Hospital, Goyang-Si, South Korea; 89 Assistance Publique Hôpitaux de Paris, Hopital Saint Antoine, Paris, France

**Keywords:** Comparative genomics, Molecular neuroscience

## Abstract

Tourette syndrome (TS) is a neuropsychiatric disorder of complex genetic architecture involving multiple interacting genes. Here, we sought to elucidate the pathways that underlie the neurobiology of the disorder through genome-wide analysis. We analyzed genome-wide genotypic data of 3581 individuals with TS and 7682 ancestry-matched controls and investigated associations of TS with sets of genes that are expressed in particular cell types and operate in specific neuronal and glial functions. We employed a self-contained, set-based association method (SBA) as well as a competitive gene set method (MAGMA) using individual-level genotype data to perform a comprehensive investigation of the biological background of TS. Our SBA analysis identified three significant gene sets after Bonferroni correction, implicating ligand-gated ion channel signaling, lymphocytic, and cell adhesion and transsynaptic signaling processes. MAGMA analysis further supported the involvement of the cell adhesion and trans-synaptic signaling gene set. The lymphocytic gene set was driven by variants in *FLT3*, raising an intriguing hypothesis for the involvement of a neuroinflammatory element in TS pathogenesis. The indications of involvement of ligand-gated ion channel signaling reinforce the role of GABA in TS, while the association of cell adhesion and trans-synaptic signaling gene set provides additional support for the role of adhesion molecules in neuropsychiatric disorders. This study reinforces previous findings but also provides new insights into the neurobiology of TS.

## Introduction

Tourette syndrome (TS) is a chronic neurodevelopmental disorder characterized by several motor tics and at least one vocal tic that persist more than a year^1^. Its prevalence is between 0.6 and 1% in school-aged children^2,3^. Although TS is highly polygenic in nature, it is also highly heritable^4^. The population-based heritability is estimated at 0.7^5,6^, with SNP-based heritability ranging from 21 to 58%^4^ of the total. The genetic risk for TS that is derived from common variants is spread throughout the genome^4^. The two genome-wide association studies (GWAS) conducted to date^7,8^ suggest that TS genetic variants may be associated, in aggregate, with tissues within the cortico-striatal and cortico-cerebellar circuits, and in particular, the dorsolateral prefrontal cortex. The GWAS results also demonstrated significant ability to predict tic severity using TS polygenic risk scores^7,9^. A genome-wide CNV study identified rare structural variation contributing to TS on the *NRXN1* and *CNTN6* genes^10^. De novo mutation analysis studies in trios have highlighted two high confidence genes, *CELSR* and *WWC1*, and four probable genes, *OPA1*, *NIPBL*, *FN1*, and *FBN2* to be associated with TS^11,12^.

Investigating clusters of genes, rather than relying on single-marker tests is an approach that can significantly boost power in a genome-wide setting^13^. Common variant studies can account for a substantial proportion of additive genetic variance^14^ and have indeed produced a wealth of variants associated with neuropsychiatric disorders, which, however, lack strong predictive qualities, an issue commonly referred to as “missing heritability”^15^. Theoretical, as well as empirical, observations have long hinted toward the involvement of non-additive genetic variance into the heritability of common phenotypes. As such, pathway analyses could pave the way toward the elucidation of missing heritability in complex disease.

This approach has already proven useful in early genome-wide studies of TS. The first published TS GWAS, which included 1285 cases and 4964 ancestry-matched controls did not identify any genome-wide significant loci. However, by partitioning functional- and cell-type-specific genes into gene sets, an involvement of genes implicated in astrocyte carbohydrate metabolism was observed, with a particular enrichment in astrocyte-neuron metabolic coupling^16^. Here, we investigated further the pathways that underlie the neurobiology of TS, performing gene set analysis on a much larger sample of cases with TS and controls from the second wave TS GWAS. We employed both a competitive gene set analysis as implemented through MAGMA, as well as a self-contained analysis through a set-based association method (SBA). Besides highlighting a potential role for neuroimmunity, our work also provides further support for previously implicated pathways including signaling cascades and cell adhesion molecules.

## Materials and methods

### Samples and quality control

The sample collection and single variant analyses for the data we analyzed have been extensively described previously^7,8^. IRB approvals and consent forms were in place for all data collected and analyzed as part of this project. For the purposes of our analysis, we combined 1285 cases with TS and 4964 ancestry-matched controls from the first wave TS GWAS, with 2918 TS cases and 3856 ancestry-matched controls from the second wave TS GWAS. Standard GWAS quality control procedures were employed^17,18^. The data were partitioned first by genotyping platform and then by ancestry. The sample call rate threshold was set to 0.98, and the inbreeding coefficient threshold to 0.2. A marker call rate threshold was defined at 0.98, case-control differential missingness threshold at 0.02, and Hardy–Weinberg equilibrium (HWE) threshold to 10^−6^ for controls and 10^−10^ for cases. Before merging the partitioned datasets, we performed pairwise tests of association and missingness between the case-only and control-only subgroups to address potential batch effect issues. All SNPs with *p*-values ≤10^−06^ in any of these pairwise quality control analyses were removed. After merging all datasets, principal component analysis was utilized to remove samples that deviated more than 6 standard deviations and to ensure the homogeneity of our samples in the ancestry space of the first 10 principal components, through the use of the EIGENSOFT suite^19^. Identity-by-descent analysis with a threshold of 0.1875 was used to remove related samples, and thus to avoid confounding by cryptic relatedness. After quality control, the final merged dataset consisted of 3581 cases with TS and 7682 ancestry-matched controls on a total of 236,248 SNPs, annotated using dbSNP version 137 and the hg19 genomic coordinates.

We assessed the genomic variation in our data through PCA analysis to identify potential population structure (Supplementary Fig. 1 and Supplementary Table 1). The variation in our data was reduced to a triangular shape in the two-dimensional space of the first two principal components. One tip was occupied by Ashkenazi Jewish samples, the second by the Southern European samples, and the other by the North Europeans. Depicting geography, the Southern to Nothern axis was populated by European-ancestry samples. The first five principal components were deemed statistically significant (Tracy Widom test as implemented by EIGENSOFT, Supplementary Table 1) and were added to the association model as covariates, in order to avoid population structure influencing our results.

### Gene sets

We collected neural-related gene sets from multiple studies on pathway analyses in neuropsychiatric disorders^16,20–24^. These studies relied on an evolving list of functionally-partitioned gene sets, focusing mainly on neural gene sets, including synaptic, glial sets, and neural cell-associated processes. We added a lymphocytic gene set also described in these studies^23^, in order to also investigate potential neuroimmune interactions.

In total, we obtained 51 gene sets, which we transcribed into NCBI Entrez IDs and subsequently filtered by removing gene sets that contained fewer than 10 genes. Forty-five gene sets fit our criteria and were used to conduct the analyses.

We examined two primary categories of pathway analysis methods, the competitive 25 and the self-contained test^16^^,^^25^. The competitive test compares the association signal yielded by the tested gene set to the association signals that do not reside in it^26,27^. In this type of test, the null hypothesis is that the tested gene set attains the same level of association with disease as equivalent random gene sets. In contrast, the self-contained test investigates associations of each tested gene set with the trait, and not with other gene sets, meaning that the null hypothesis in this case is that the genes in the gene set are not associated with the trait^25,27^. Therefore, for a competitive test, there should be data for the whole breadth of the genome, but this test cannot provide information regarding how strongly the gene set is associated with the trait^28^. We employ both methods for a comprehensive investigation into the neurobiological background of TS.

### MAGMA on raw genotypes

We ran MAGMA^26^ on the individual-level genotype data using the aforementioned filtered gene set lists. MAGMA performs a three-step analytic process. First, it annotates the SNPs by assigning them to genes, based on their chromosomal location. Then it performs a gene prioritization step, which is used to perform the final gene set analysis step. We used a genomic window size of ±10 kb and the top 5 principal components as covariates to capture population structure. SNP-to-gene assignments were based on the NCBI 37.3 human gene reference build. The number of permutations required for the analysis was determined by MAGMA, using an adaptive permutation procedure leading to 11,263 permutations. MAGMA employs a family-wise error correction calculating a significance threshold of 0.00100496.

### Set-based association (SBA) test

We conducted SBA tests on the raw individual genotype data, as described in PLINK^25,29^ and adapted in a later publication^30^. This test relies on the assignment of individual SNPs to a gene, based on their position, and thus to a pathway, according to the NCBI 37.3 human gene reference build. After single-marker association analysis, the top LD-independent SNPs from each set are retained and selected in order of decreasing statistical significance, and the mean of their association *p*-values is calculated. We permuted the case/control status, repeating the previous association and calculation steps described above, leading to the empirical *p*-value for each set. The absolute minimum number of permutations required for crossing the significance level is dictated by the number of gene sets tested. Testing for 45 gene sets requires at least 1000 permutations to produce significant findings. PLINK’s max(t) test recommends at least 64,000 permutations. We opted to increase the number of permutations to one million, the maximum that was computationally feasible, to maximize our confidence in the outcomes, given our large sample size.

We used logistic regression as the association model on the genotypes and the first five principal components as covariates on the genotype data to conduct the SBA test with the collected neural gene sets. Another repetition of this step was performed with a simple association test, to test for this method’s robustness to population structure. We proceeded to run the analysis on all samples, using all gene sets at a 10 kb genomic window size, the first five principal components as covariates, and one million permutations. Since the permutations were performed on the phenotypic status of the samples, and only served as a method of association of the trait with the gene sets, we also corrected the results by defining the significance threshold through Bonferroni correction at 1.1 × 10^−3^ (0.05/45).

## Results

For the gene set association analysis, we ran PLINK’s self-contained set-based association method and MAGMA’s competitive association method, using the same 45 gene sets on the processed genotyped data of 3581 cases and 7682 ancestry-matched controls on a total of 236,248 SNPs. By performing both methods of analysis we aimed to obtain a global assessment of the gene sets’ relationship with TS.

**Fig. 1:**
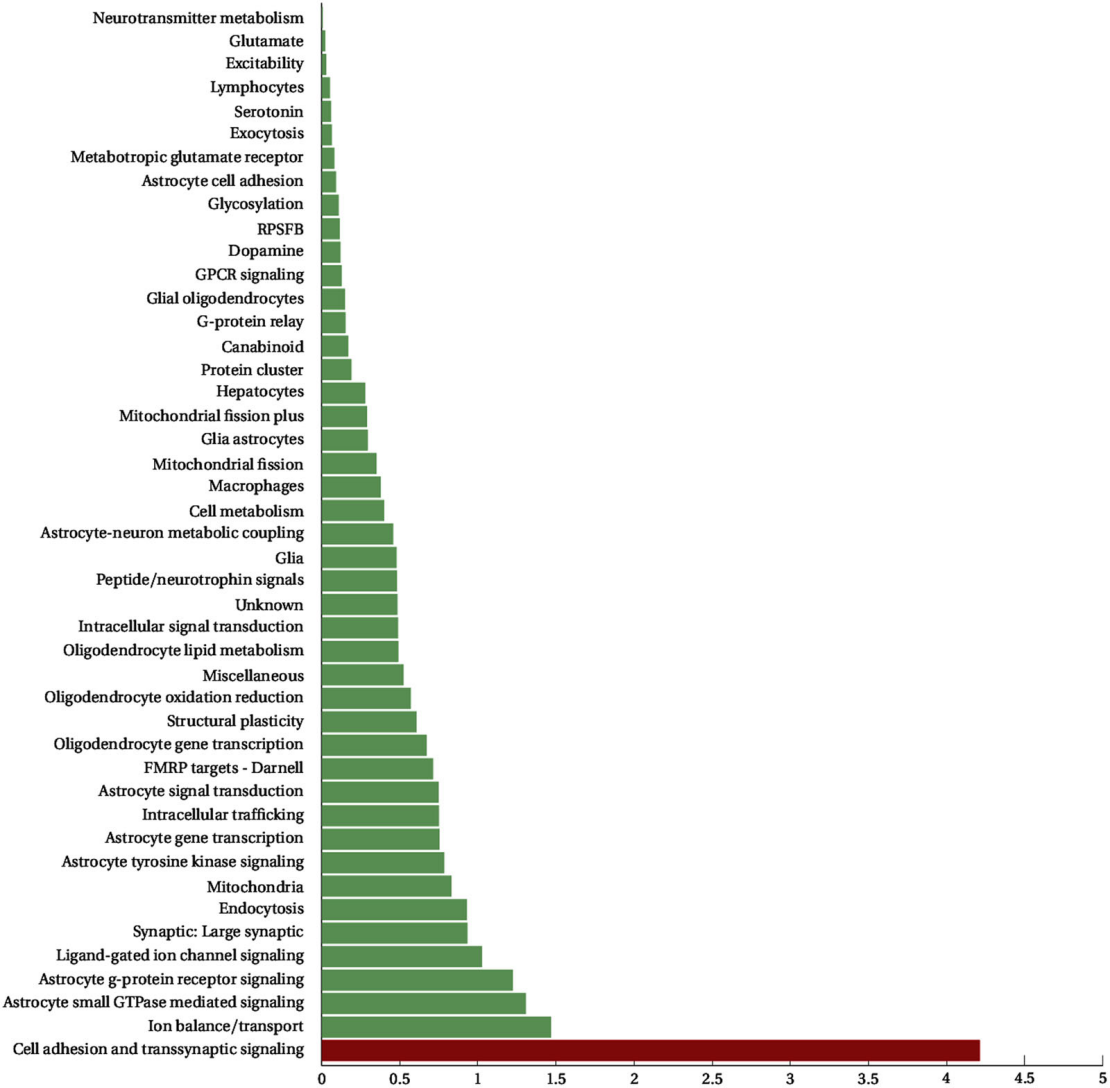
Results of gene set analysis as implemented by MAGMA. The gene set that crossed the significance threshold is depicted in red.

MAGMA analysis identified one significant gene set (Fig. 1), cell adhesion and trans-synaptic signaling (CATS), which achieved a nominal *p*-value of 6.2 × 10^−5^ (permuted *p*-value of 0.0032). While the CATS gene set is comprised of 83 genes, MAGMA’s annotation step prioritized 72 of its genes for the gene set analysis. It involves 3290 variants that were reduced to 1627 independent variants in our data. Results were mainly driven by associations in the CDH26, CADM2, and OPCML genes as indicated by MAGMA gene-based analysis (Table 1). In the gene-based tests, CDH26 attained a *p*-value of 8.9526 × 10^−6^, CADM2 a *p*-value of 4.6253 × 10^−4^, and OPCML a *p*-value of 7.9851 × 10^−4^, neither crossing the genome-wide significance threshold for gene tests (2.574 × 10^−6^ calculated on 19,427 genes contained in the NCBI 37.3 version of RefGene).

**Table 1:** Statistically significant result of MAGMA gene set analysis.

Gene set							Genes	*P*-value	*P*_corr_
Cell adhesion and transsynaptic signaling							72	6.1736e−05	0.00318
Gene ID	Chr	Start	End	SNPs	Param	*N*	Z-stat	*P*-value	Gene name
60437	20	58528471	58593772	4	3	11263	4.2895	8.95e−06	Cadherin 26 (CDH26)
253559	3	85003133	86128579	42	18	11263	3.3124	0.00046	Cell Adhesion molecule 2 (CADM2)
4978	11	132279875	133407403	210	106	11263	3.1564	0.00079	Opioid binding protein/cell adhesion molecule like (OPCML)
1007	5	26875709	27043689	14	7	11263	2.9627	0.0015	Cadherin 9 (CDH9)
4685	21	22365633	22918892	61	29	11263	2.7975	0.0025	Neural Cell adhesion molecule 2 (NCAM2)
961	3	107756941	107814935	6	4	11263	2.6465	0.0040	CD47 molecule (CD47)
1003	16	66395525	66443689	11	6	11263	2.0242	0.021	Cadherin 5 (CDH5)
199731	19	44121519	44148991	4	3	11263	1.984	0.023	CADM4 (cell adhesion molecule 4)
708	17	5331099	5347471	1	1	11263	1.9269	0.026	C1QBP (complement C1q binding protein)
2017	11	70239612	70287690	2	2	11263	1.8709	0.030	CTTN (cortactin)
4045	3	115516210	116169385	56	29	11263	1.8095	0.035	Limbic system-associated membrane protein (LSAMP)
8502	2	159308476	159542941	19	9	11263	1.7503	0.040	Plakophilin 4 (PKP4)
5097	5	141227655	141263361	3	3	11263	1.6903	0.045	PCDH1 (protocadherin 1)
26047	7	145808453	148123090	237	110	11263	1.6621	0.048	Contactin associated protein-like 2 (CNTNAP2)
4155	18	74685789	74849774	49	30	11263	1.6502	0.049	MBP (maltose-binding protein)

The cell adhesion and transsynaptic signaling gene set achieved statistical significance. Genes within this set that achieved nominal significance with gene-based test implemented by MAGMA are also listed here. Gene ID refers to Entrez ID, Param to the number of SNPs used for the SNP-wise analysis.

**Fig. 2:**
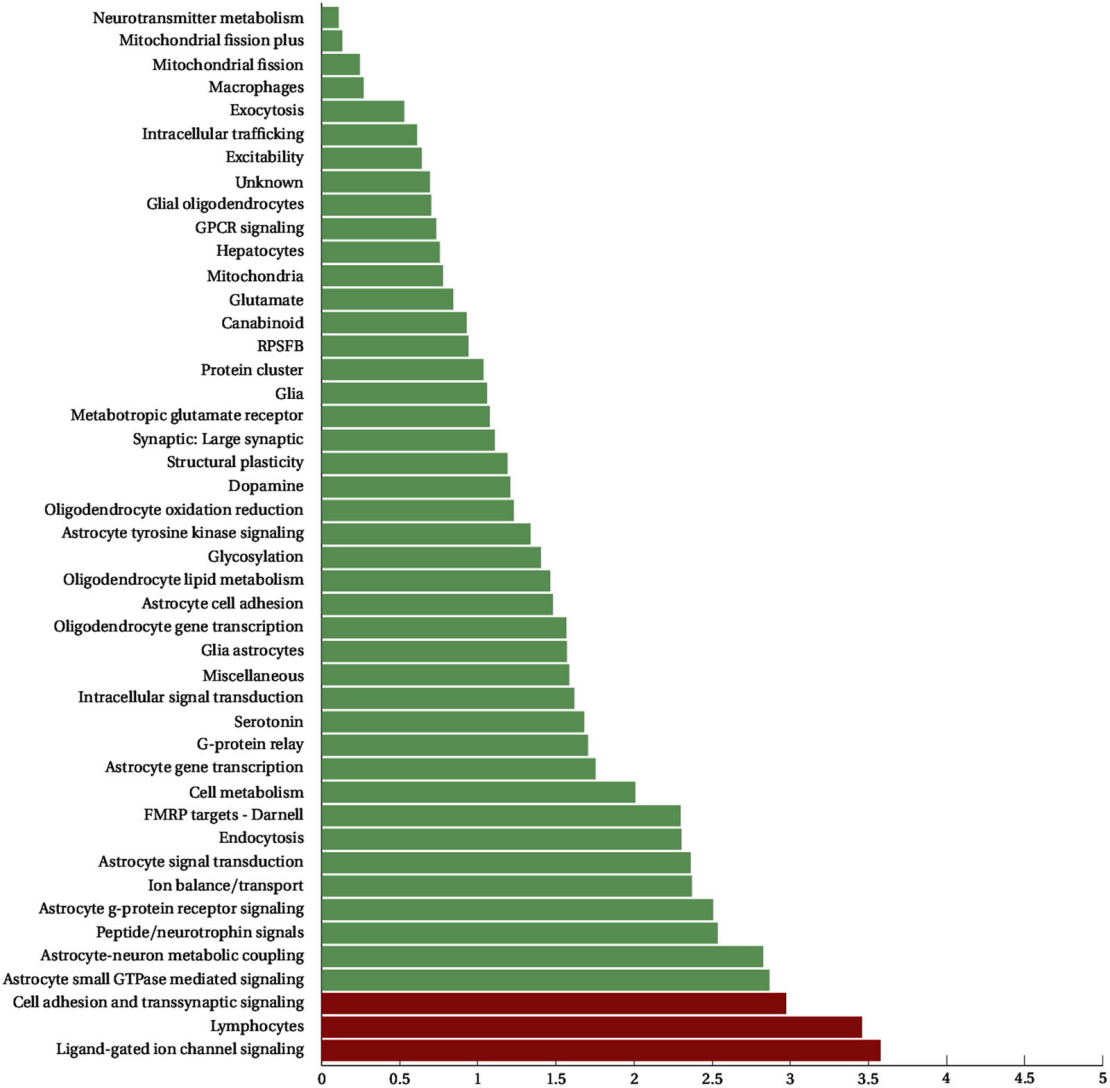
Results of gene set analysis as implemented by SBA. The gene sets that crossed the significance threshold are depicted in red.

We next run SBA, which conducts an initial single-marker association step before performing permutations to calculate empirical *p*-values for the gene sets. This association step is performed on the total number of variants that are associated with the genes involved in the gene sets, leading to a subset of 25,630 variants in our data, which are then filtered based on their LD. Analysis identified three gene sets as significant (Table 2), the ligand-gated ion channel signaling (LICS) (P: 2.67 × 10^−4^), the lymphocytic (P: 3.5 × 10^−4^), and the cell adhesion and trans-synaptic signaling (CATS) (P: 1.07 × 10^−3^). Detailed results for all the tested gene sets are shown in Fig. 2.

**Table 2:** Statistically significant results of the SBA analysis.

Gene set						SNPs	NSIG	ISIG	EMP1
Chr	SNP	BP	A1	F_A	F_U	A2	*P*	OR	Genes implicated
Ligand-gated ion channel signaling						683	66	5	0.000267
4	rs1391174	46072596	T	0.4892	0.4586	C	1.764e−05	1.131	GABRG1(0)
5	rs9790873	45291514	C	0.1535	0.1335	T	5.621e−05	1.177	HCN1(0)
9	rs2259639	101317401	T	0.2751	0.2982	C	0.0003612	0.8928	GABBR2(0)
9	rs1930415	101238974	T	0.2218	0.2424	C	0.0007006	0.8908	GABBR2(0)
11	rs949054	120795888	C	0.2241	0.2053	T	0.001281	1.118	GRIK4(0)
Lymphocytes						799	50	5	0.00035
19	rs16986092	55433696	T	0.1158	0.09473	C	1.093e−06	1.251	NCR1(+9.257 kb)|NLRP7(−1.18 kb)
13	rs1933437	28624294	G	0.4183	0.3871	A	8.482e−06	1.138	FLT3(0)
3	rs2243123	159709651	C	0.2515	0.2759	T	0.0001167	0.8817	IL12A(0)|IL12A-AS1(0)
7	rs3801983	18683672	C	0.1928	0.2133	T	0.0003981	0.8808	HDAC9(0)
5	rs2230525	66478626	C	0.08431	0.07127	T	0.0005641	1.2	CD180(0)
Cell adhesion and transsynaptic signaling						3290	292	5	0.00107
20	rs1002762	58580885	G	0.2305	0.2028	A	2.031e−06	1.178	CDH26(0)
21	rs2826825	22762779	G	0.376	0.3487	A	6.698e−05	1.126	NCAM2(0)
11	rs7925725	131449365	C	0.3709	0.3979	A	0.0001099	0.8921	NTM(0)
11	rs12224080	131816849	G	0.09841	0.08353	A	0.0002519	1.198	NTM(0)
3	rs6773575	77060574	C	0.0964	0.1126	A	0.000256	0.8407	ROBO2(0)

Three pathways achieved significance. Association statistics for the top five SNPs driving the signal in each set are also shown. NSIG is the number of SNPs crossing the nominal significance threshold. EMP1 is the empirical *p*-value attained by the tested gene set. *P* is the *p*-value of the original single-marker association, OR is the respective odds ratio. A1 is the minor allele and A2 the major allele. F_A and F_U are the frequencies of the minor allele in case and control samples, respectively.

The LICS gene set was the top-scoring gene set, including 38 genes and involving 683 variants, 66 of which were associated with TS. The gene set’s signal was primarily driven by variants residing in the genes of the *γ*-aminobutyric acid receptors *GABRG1* and *GABBR2*, the *HCN1* channel gene and the glutamate receptor gene *GRIK4*. This signal was driven primarily by an association with SNP rs9790873, which is an eQTL for *HCN1* in tibial nerve, according to GTEx^31^. *GABBR2* is represented by two top SNPs, that are LD-independent, and removing either of those SNPs from the gene set did not cause the gene set to drop under the significance threshold.

The lymphocytic gene set was the next top-scoring gene set, including 143 genes that translated to 799 variants in our data, with 50 of these variants associated with TS. Its signal was driven by a missense variant inside the *FLT3* gene and an intergenic variant between *NCR1* and *NLRP7*, followed by *IL12A*, *HDAC9*, *CD180*. The rs1933437 SNP is the top variant for *FLT3*, and is a possibly damaging missense variant^32^, located in the sixth exon of the *FLT3* gene leading to a p.Thr227Met mutation. It is a very common variant and the sixth exon appears to be less expressed than downstream exons. Given the tissues in which this eQTL affects *FLT3*’s expression, we tested the lymphocytic gene set by removing *FLT3* from it, to identify whether the lymphocytic gene set association was biased by the presence of *FLT3*. After removing *FLT3*, the lymphocytic gene set association statistic decreased slightly (P: 0.00012), driven mainly by *NCR1*/*NRLP7*.

The third significant gene set, CATS, consisted of 83 genes, including multiple large genes. CATS was identified by both SBA and MAGMA in our analyses, and both gene set approaches identified *CDH26* as the gene with the lowest *p*-value. Both SBA and MAGMA also identified *NCAM2*, *NTM*, and *ROBO2* as strongly associated with TS, with *NTM* represented by two LD-independent SNPs. CATS’s top SNP, rs1002762, resides in the *CDH26* gene on chromosome 20, and is the top associated SNP in our data (P: 2.031 × 10^−6^) with an odds ratio of 1.178.

Notable results from the SBA also include the Astrocyte small GTPase mediated signaling (ASGMS) and the Astrocyte-neuron metabolic coupling (ANMC) gene sets, with a *p*-values slightly under the significance thresholds. These gene sets attained a *p*-value of 0.00137 and 0.001504, respectively.

## Discussion

Seeking to elucidate the neurobiology of TS, we present here the largest study to date aiming to interrogate the involvement of sets of genes that are related to neuronal and glial function in TS. We analyzed data from our recently performed TS GWAS and conducted two distinct types of testing, a competitive, regression-based test (MAGMA) and a self-contained, *p*-value combining test (SBA). Self-contained tests investigate for associations with a phenotype, while competitive tests compare a specific gene set against randomly generated gene sets. We employed both methods to perform a comprehensive investigation of the biological background of TS.

A potential problem in pathway analysis is false SNP assignment to genes, which in turn may increase false results. In order to address this issue, most studies in the literature use short window sizes (10–20 kb) when assigning SNPs to genes. Here, we used a 10 kb window, paired with excessive permutations to avoid false assignments, that would introduce false positive results. There is evidence that long-range SNP effects could play a role, mostly associated with large insertion/deletion events that are not in the scope of this study and would likely hamper the analysis^33^.

MAGMA’s regression-based algorithm has been reported to account for gene size biases, as can be also discerned by the variable sizes of the top genes. MAGMA’s top prioritized gene, *CDH26*, is represented by 4 SNPs in our data, *CADM2* by 42, while *OPCML* is represented by 210 SNPs, as it covers an extensive genomic region. We addressed such issues in SBA by setting a low *r*^2^ threshold and conditioning on any LD-independent SNPs that resided on the same gene.

The gene sets used in our study come from a line of function-based analyses, aiming to investigate neurobiological mechanisms in neuropsychiatric disorders. A previous pathway analysis using individual-level genotype data of the first wave TS GWAS identified genes involved in astrocytic-neuron metabolic coupling, implicating astrocytes in TS pathogenesis^16^. In this study, we took advantage of the increased sample size of the second wave TS GWAS and the mechanics of the two distinct methods to identify gene sets associated with TS that provide a novel insight into the pathogenesis of TS, and substantiate the role of neural processes in this neuropsychiatric disorder.

The ANMC gene set that contains genes involved in carbohydrate metabolism in astrocytes was the single identified gene set in the previous pathway analysis study on TS^16^, raising a hypothesis on a potential mechanism that involves altered metabolism of glycogen and glutamate/*γ*-aminobutyric acid in the astrocytes. In our study, the ANMC gene set scored slightly under the significance threshold.

Here, analyzing a much larger sample size we identified three sets of genes as significantly associated to the TS phenotype. Among them the LICS gene set, which involves genes implicated in ion channel signaling through *γ*-aminobutyric acid and glutamate. Several genes in the LICS gene set have been previously implicated in neuropsychiatric phenotypes. *HCN1*, a hyperpolarization-activated cation channel involved in native pacemaker currents in neurons and the heart, has been significantly associated with schizophrenia and autism^34–36^. *GABRG1*, an integral membrane protein that inhibits neurotransmission by binding to the benzodiazepine receptor, has yielded mild associations with general cognitive ability^37^ and epilepsy^38^, while *GABBR2*, a g-protein-coupled receptor that regulates neurotransmitter release, with schizophrenia^39^ and post-traumatic stress disorder^40^ in multiple studies. The GABA-ergic pathway has been previously implicated in TS, and recent advances showcased the possibility that a GABA-ergic transmission deficit can contribute toward TS symptoms^41^. *GRIK4*, encoding a glutamate-gated ionic channel, has shown associations with mathematical ability and educational attainment^42^ and weaker associations with attention-deficit hyperactivity disorder^43^. The *γ*-aminobutyric acid receptors and the HCN channel, are features of inhibitory interneurons^44^ and also identified in the brain transcriptome of individuals with TS^45^, adding to the evidence that the phenotype of TS could be influenced by an inhibitory circuit dysfunction, as has previously been proposed^46^.

Individuals with TS are reported to present elevated markers of immune activation^45,47^. In addition, a number of studies have implicated neuroimmune responses with the pathogenesis of TS^48–50^. We investigated neuroimmune interactions by interrogating association to a gene set designed by Goudriaan et al.^23^ to study enrichment in lymphocytic genes. Indeed, our analysis yielded a statistically significant signal. The *FLT3* association coincides with the results of the second wave TS GWAS, in which *FLT3* was the only genome-wide significant hit^7^. *FLT3* and its ligand, *FLT3LG*, have a known role in cellular proliferation in leukemia, and have been found to be expressed in astrocytic tumors^51^. The rs1933437 variant in *FLT3* is an eQTL in the brain cortex and the cerebellum^31^, and has also been implicated in the age at the onset of menarche^52^. Variants in *FLT3* have attained genome-wide significance in a series of studies focusing on blood attributes in populations of varying ancestry, and our current insights into its role are mostly based on these associations with blood cell counts, serum protein levels, hypothyroidism, and autoimmune disorders^52–55^.

*FLT3* could play a role in neuroinflammation as supported by its intriguing association with peripheral neuropathic pain. The inhibition of *FLT3* is reported to alleviate peripheral neuropathic pain (PNP)^56^, a chronic neuroimmune condition that arises from aberrations in the dorsal root ganglia. Cytokines and their receptors have been at the epicenter of the neuroimmune interactions, with microglia contributing significantly to chronic phenotypes of such states^57^. *FLT3* is a critical component for neuroimmune interactions, especially in the case of the development and sustenance of the PNP phenotype. Interestingly, pain follows sex-specific routes, with glia having a prominent role for pain propagation in males, while females involve adaptive immune cells instead^58^. These, paired with previous evidence of glial involvement in TS^16^, raise an interesting hypothesis for TS symptom sustenance, since *FLT3* has been shown to be critical for the chronicity of neuronal dysregulations^56^.

Notably, *FLT3* has a prominent role in the hematologic malignancies, with one-third of adult acute myeloid leukemia (AML) patients presenting with activating mutations in *FLT3*, and wild-type *FLT3* being found overexpressed in hematologic malignancies. *FLT3* is implicated in apoptotic mechanisms, with its mutations being associated with^59^ Warburg effect promotion, inhibition of ceramide-dependent mitophagy^60^, and induction of pro-survival signals, through downstream signaling cascades, including PI3K-Akt-mTOR, Ras/MAPK, and JAK-STAT. This mitochondrial role of *FLT3* has been further reinforced by findings that associate it with increased post-transcriptional methylation of mitochondrial tRNAs in cancer^61^. As such, *FLT3* is regarded a molecular target for therapeutic intervention^62^.

*FLT3* is expressed in the cerebellum and whole blood, while *FLT3*’s top variant, rs1933437, is an eQTL for *FLT3* on GTEx^31^ in various brain tissues, such as the cortex, the cerebellum, the hypothalamus, the frontal cortex (BA9), and non-brain tissues, such as the skin, the pancreas, and adipose tissues. In order to test the robustness of the lymphocytic association in our findings, we repeated the analysis after removing *FLT3* from the lymphocytic gene set. The *p*-value of the gene set decreased, but still remained significant, due to the association in the *NCR1/NLRP7* locus. Besides *FLT3*, the other genes included in this gene set are also quite intriguing to consider as potential candidates that could underlie the pathophysiology of TS. In the same vein with *FLT3*, common variants in *NCR1* have also been significantly associated with blood protein levels^63^. HDAC9 has been significantly associated with androgenetic alopecia^52,64^, hair color^52^, and ischemic stroke^65^. These seem to follow previous knowledge, given that genes involved in ischemic stroke have been identified as a common component between TS and ADHD^66^, and that TS, similar to other neuropsychiatric disorders, demonstrates a distinct preference for males. CD180 has shown associations with general cognitive ability^37^.

The CATS gene set involves many cell adhesion molecules, with the top signals found in *CDH26*. CDH26 is a cadherin that regulates leukocyte migration, adhesion, and activation, especially in the case of allergic inflammation^67^. Cell adhesion molecules have been consistently implicated in phenotypes related to brain function, with the latest addition of the high confidence TS gene *CELSR3*, a flamingo cadherin, that was identified in a large scale de novo variant study for TS^12^. Their relation to TS has been well documented, with the notable examples of neurexins, contactins, neuroligins, and their associated proteins^10,68–70^. These genes were present in the CATS gene set but did not reach a level of significance in our analysis. This hints toward their possible involvement in TS mostly through rare variants^10,68,69^, a notion reinforced by findings in other neuropsychiatric disorders^71,72^.

Most of the genes contained in the identified gene sets in this study are involved in cognitive performance, mathematical ability, and educational attainment^42^. OPCML, CADM2, and ROBO2 have been implicated in neuromuscular and activity phenotypes, such as grip strength^73^, physical activity^74^, and body mass index^52^. ROBO2 has been associated with depression^75^, expressive vocabulary in infancy^76^, while CADM2 is associated to a multitude of phenotypes, including anxiety^75^, risk-taking behavior, and smoking^77^. NTM displays similar patterns of pleiotropy, associated with smoking^52^, myopia^64^, hair color^78^, anxiety^75^, asperger’s syndrome^79^, bipolar disorder with schizophrenia^80^, and eating disorders^81^. NCAM2 and NTM, similarly to the lymphocytic genes, have been significantly associated with blood protein levels^82^ and leukocyte count^52^, respectively. Many of these phenotypes are known TS comorbidities, presenting themselves commonly or less commonly in TS cases, and others are related to functions that get impaired in TS symptomatology.

The CATS gene set was identified in both methods indicating the involvement of cell adhesion molecules in transsynaptic signaling. Using genotypes with both methods as a means of identifying pathways instead of summary statistics, gave our study the edge of sample-specific linkage disequilibrium rather than relying on an abstract linkage disequilibrium pattern reference. Our current understanding for regional structures of the genome and the *cis*-effects of genomic organization will aid the refinement of these associations as well as help shape our understanding of the pleiotropic mechanisms in the identified loci potentially responsible for disease pathogenesis.

In conclusion, our analysis reinforces previous findings related to TS neurobiology while also providing novel insights: We provide further support for the role of *FLT3* in TS, as well as the possibility for the involvement of the GABA-ergic biological pathway in TS pathogenesis. At the same time, our study highlights the potential role of glial-derived neuroimmunity in the neurobiology of TS opening up intriguing hypotheses regarding the potential for gene-environment interactions that may underlie this complex phenotype.

## Supplementary information

Supplemental Material

## References

[1] Robertson MM, Cavanna AE, Eapen V (2015). Gilles de la Tourette syndrome and disruptive behavior disorders: prevalence, associations, and explanation of the relationships. J. Neuropsychiatry Clin. Neurosci..

[2] Scharf JM (2015). Population prevalence of Tourette syndrome: a systematic review and meta-analysis. Mov. Disord..

[3] Robertson MM, Eapen V, Cavanna AE (2009). he international prevalence, epidemiology, and clinical phenomenology of Tourette syndrome: a cross-cultural perspective. J. Psychosom. Res..

[4] Davis, L. K. Partitioning the heritability of Tourette syndrome and obsessive compulsive disorder reveals differences in genetic architecture. *PLoS Genetics* **9**, e1003864 (2013). 10.1371/journal.pgen.1003864 PMC3812053 24204291

[5] Robertson MM (2017). Gilles de la Tourette syndrome. Nat. Rev. Dis. Prim..

[6] Mataix-Cols D (2015). Familial risks of Tourette syndrome and chronic tic disorders: a population-based cohort study. JAMA Psychiatry.

[7] Yu, D. Interrogating the genetic determinants of Tourette syndrome and other tic disorders through genome-wide association studies. *Am. J. Psychiatry* **176**, 217–227 (2019). 10.1176/appi.ajp.2018.18070857 PMC6677250 30818990

[8] Scharf JM (2013). Genome-wide association study of Tourette’s syndrome. Mol. Psychiatry.

[9] Abdulkadir M (2019). Polygenic risk scores derived from a Tourette syndrome genome-wide association study predict presence of tics in the Avon Longitudinal Study of Parents and Children cohort. Biol. Psychiatry.

[10] Huang, A. Rare copy number variants in NRXN1 and CNTN6 increase risk for Tourette syndrome. *Neuron* **94**, 1101–1111 (2017). 10.1016/j.neuron.2017.06.010 PMC5568251 28641109

[11] Willsey AJ (2017). De novo coding variants are strongly associated with Tourette disorder. Neuron.

[12] Wang S (2018). De novo sequence and copy number variants are strongly associated with Tourette disorder and implicate cell polarity in pathogenesis. Cell Rep..

[13] Ballard DH, Cho J, Zhao H (2010). Comparisons of multi-marker association methods to detect association between a candidate region and disease. Genet. Epidemiol..

[14] Visscher, P. M. 10 Years of GWAS discovery: biology, function, and translation. *Am. J. Hum. Genet.* **101**, 5–22 (2017). 10.1016/j.ajhg.2017.06.005 PMC5501872 28686856

[15] Zuk O (2014). Searching for missing heritability: designing rare variant association studies. Proc. Natl. Acad. Sci. USA.

[16] de Leeuw C (2015). Involvement of astrocyte metabolic coupling in Tourette syndrome pathogenesis. Eur. J. Hum. Genet..

[17] Turner, S. Quality control procedures for genome-wide association studies. *Curr. Protoc. Hum. Genet.* **68**, 1.19.1–1.19.18 (2011). 10.1002/0471142905.hg0119s68 PMC3066182 21234875

[18] Lam, M. RICOPILI: rapid imputation for COnsortias PIpeLIne. *Bioinformatics* **36**, 930–933 (2020). 10.1093/bioinformatics/btz633 PMC7868045 31393554

[19] Price AL (2006). Principal components analysis corrects for stratification in genome-wide association studies. Nat. Genet..

[20] Ruano D (2010). Functional gene group analysis reveals a role of synaptic heterotrimeric g proteins in cognitive ability. Am. J. Hum. Genet..

[21] Lips ES (2012). Functional gene group analysis identifies synaptic gene groups as risk factor for schizophrenia. Mol. Psychiatry.

[22] Duncan, L. E. Pathway analyses implicate glial cells in schizophrenia. *PLoS ONE* **9**, e89441 (2014). 10.1371/journal.pone.0089441 PMC3933626 24586781

[23] Goudriaan A (2014). Specific glial functions contribute to Schizophrenia susceptibility. Schizophrenia Bull..

[24] Jansen A (2017). Gene-set analysis shows association between FMRP targets and autism spectrum disorder. Eur. J. Hum. Genet..

[25] Purcell S (2007). PLINK: a tool set for whole-genome association and population-based linkage analyses. Am. J. Hum. Genet..

[26] de Leeuw, C. A., Mooij, J. M., Heskes, T. & Posthuma, D. MAGMA: generalized gene-set analysis of GWAS data. *PLoS Comput. Biol.* **11**, e1004219 (2015). 10.1371/journal.pcbi.1004219 PMC4401657 25885710

[27] Goeman JJ, Buhlmann P (2007). Analyzing gene expression data in terms of gene sets: methodological issues. Bioinformatics.

[28] Mooney MA, Wilmot B (2015). Gene set analysis: a step-by-step guide. Am. J. Med. Genet. Part B: Neuropsychiatr. Genet..

[29] Chang CC (2015). Second-generation PLINK: rising to the challenge of larger and richer datasets. GigaScience.

[30] Skafidas E (2014). Predicting the diagnosis of autism spectrum disorder using gene pathway analysis. Mol. Psychiatry.

[31] Carithers LJ (2015). A novel approach to high-quality postmortem tissue procurement: the GTEx project. Biopreservation Biobanking.

[32] Karczewski, K. J. The mutational constraint spectrum quantified from variation in 141,456 humans. *Nature* **581**, 434–443 (2020). 10.1038/s41586-020-2308-7 PMC7334197 32461654

[33] Brodie A, Azaria JR, Ofran Y (2016). How far from the SNP may the causative genes be?”. Nucleic Acids Res..

[34] Pardiñas AF (2018). Common schizophrenia alleles are enriched in mutation-intolerant genes and in regions under strong background selection. Nat. Genet..

[35] Autism Spectrum Disorders Working Group of The Psychiatric Genomics Consortium. (2017). Meta-analysis of GWAS of over 16,000 individuals with autism spectrum disorder highlights a novel locus at 10q24.32 and a significant overlap with schizophrenia. Mol. Autism.

[36] Okbay A (2016). Genome-wide association study identifies 74 loci associated with educational attainment. Nature.

[37] Davies G (2018). Study of 300,486 individuals identifies 148 independent genetic loci influencing general cognitive function. Nat. Commun..

[38] International League Against Epilepsy Consortium on Complex Epilepsies. (2014). Genetic determinants of common epilepsies: a meta-analysis of genome-wide association studies. Lancet Neurol..

[39] Ikeda, M. Genome-Wide Association Study Detected Novel Susceptibility Genes for Schizophrenia and Shared Trans-Populations/Diseases Genetic Effect. *Schizophr. Bull.* (2018). 10.1093/schbul/sby140 PMC6581133 30285260

[40] Xie P (2013). Genome-wide association study identifies new susceptibility loci for posttraumatic stress disorder. Biol. Psychiatry.

[41] Puts NAJ (2015). Reduced GABAergic inhibition and abnormal sensory symptoms in children with Tourette syndrome. J. Neurophysiol..

[42] Lee JJ (2018). Gene discovery and polygenic prediction from a genome-wide association study of educational attainment in 1.1 million individuals. Nat. Genet..

[43] Yang L (2013). Polygenic transmission and complex neuro developmental network for attention deficit hyperactivity disorder: genome-wide association study of both common and rare variants. Am. J. Med. Genet. Part B, Neuropsychiatr. Genet..

[44] Kelsom C, Lu W (2013). Development and specification of GABAergic cortical interneurons. Cell Biosci..

[45] Lennington JB (2016). Transcriptome analysis of the human striatum in Tourette syndrome. Biol. Psychiatry.

[46] Rapanelli M, Frick LR, Pittenger C (2017). The role of interneurons in autism and Tourette syndrome. Trends Neurosci..

[47] Krause DL, Müller N (2012). The relationship between Tourette’s syndrome and infections. Open Neurol. J..

[48] Ercan-Sencicek AG (2010). L-histidine decarboxylase and Tourette’s syndrome. N. Engl. J. Med..

[49] Castellan L (2014). Histidine decarboxylase deficiency causes Tourette syndrome: parallel findings in humans and mice. Neuron.

[50] Alexander J (2016). Targeted re-sequencing approach of candidate genes implicates rare potentially functional variants in Tourette syndrome etiology. Front. Neurosci..

[51] Eßbach C (2013). Abundance of Flt3 and its ligand in astrocytic tumors. OncoTargets Ther..

[52] Kichaev G (2019). Leveraging polygenic functional enrichment to improve GWAS power. Am. J. Hum. Genet..

[53] Astle WJ (2016). The allelic landscape of human blood cell trait variation and links to common complex disease. Cell.

[54] Jain D (2017). Genome-wide association of white blood cell counts in Hispanic/Latino Americans: the Hispanic Community Health Study/Study of Latinos. Hum. Mol. Genet..

[55] Kanai M (2018). Genetic analysis of quantitative traits in the Japanese population links cell types to complex human diseases. Nat. Genet..

[56] Rivat C (2018). Inhibition of neuronal FLT3 receptor tyrosine kinase alleviates peripheral neuropathic pain in mice. Nat. Commun..

[57] Marchand F, Perretti M, McMahon SB (2005). Role of the immune system in chronic pain. Nat. Rev. Neurosci..

[58] Sorge RE (2015). Different immune cells mediate mechanical pain hypersensitivity in male and female mice. Nat. Neurosci..

[59] Ju HQ (2017). ITD mutation in FLT3 tyrosine kinase promotes Warburg effect and renders therapeutic sensitivity to glycolytic inhibition. Leukemia.

[60] Dany M (2016). Targeting FLT3-ITD signaling mediates ceramide-dependent mitophagy and attenuates drug resistance in AML. Blood.

[61] Y. Idaghdour, Y. & Hodgkinson, A. Integrated genomic analysis of mitochondrial RNA processing in human cancers. *Genome Med.* **9**, 36 (2017). 10.1186/s13073-017-0426-0 PMC5395977 28420414

[62] Daver, N., Schlenk, R. F., Russell, N. H. & Levis, M. J. Targeting FLT3 mutations in AML: review of current knowledge and evidence. *Leukemia* **33**, 299–312 (2019). 10.1038/s41375-018-0357-9 PMC6365380 30651634

[63] Sun BB (2018). Genomic atlas of the human plasma proteome. Nature.

[64] Pickrell JK (2016). Detection and interpretation of shared genetic influences on 42 human traits. Nat. Genet..

[65] Malik R (2018). Multiancestry genome-wide association study of 520,000 subjects identifies 32 loci associated with stroke and stroke subtypes. Nat. Genet..

[66] Tsetsos, F. Meta-analysis of tourette syndrome and attention deficit hyperactivity disorder provides support for a shared genetic basis. *Front. Neurosci.* **10**, 340 (2016). 10.3389/fnins.2016.00340 PMC4956656 27499730

[67] Caldwell JM (2017). Cadherin 26 is an alpha integrin-binding epithelial receptor regulated during allergic inflammation. Mucosal Immunol..

[68] Sun, N., Tischfield, J. A., King, R. A. & Heiman, G. A. Functional evaluations of genes disrupted in patients with Tourette’s disorder. *Front. Psychiatry* **7**, 11 (2016). 10.3389/fpsyt.2016.00011 PMC4746269 26903887

[69] Clarke RA, Lee S, Eapen V (2012). Pathogenetic model for Tourette syndrome delineates overlap with related neurodevelopmental disorders including autism. Transl. Psychiatry.

[70] Verkerk AJMH (2003). CNTNAP2 is disrupted in a family with Gilles de la Tourette syndrome and obsessive compulsive disorder. Genomics.

[71] Reissner C, Runkel F, Missler M (2013). Neurexins. Genome Biol..

[72] Chatterjee M, Schild D, Teunissen C (2019). Contactins in the central nervous system: role in health and disease. Wolters Kluwer Medknow Publ..

[73] Tikkanen E (2018). Biological insights into muscular strength: genetic findings in the UK Biobank. Sci. Rep..

[74] Klimentidis YC (2018). Genome-wide association study of habitual physical activity in over 377,000 UK Biobank participants identifies multiple variants including CADM2 and APOE. Int. J. Obes..

[75] Nagel M, Watanabe K, Stringer S, Posthuma D, van der Sluis S (2018). Item-level analyses reveal genetic heterogeneity in neuroticism. Nat. Commun..

[76] St Pourcain B (2014). Common variation near ROBO2 is associated with expressive vocabulary in infancy. Nat. Commun..

[77] Clifton EAD (2018). Genome-wide association study for risk taking propensity indicates shared pathways with body mass index. Commun. Biol..

[78] Morgan MD (2018). Genome-wide study of hair colour in UK Biobank explains most of the SNP heritability. Nat. Commun..

[79] Salyakina D (2010). Variants in several genomic regions associated with Asperger disorder. Autism Res..

[80] Wang K-S, Liu X-F, Aragam N (2010). A genome-wide meta-analysis identifies novel loci associated with schizophrenia and bipolar disorder. Schizophrenia Res..

[81] Cornelis MC (2016). A genome-wide investigation of food addiction. Obesity.

[82] Emilsson V (2018). Co-regulatory networks of human serum proteins link genetics to disease. Science.

