# Supplementary material for: Synaptic processes and immune-related pathways implicated in Tourette syndrome
Source: Transl Psychiatry. 2021 Jan 18;11:56. doi: 10.1038/s41398-020-01082-z (PMC7814139; doi:10.1038/s41398-020-01082-z)
Supplement: Supplementary file 1 — Supplemental Material [file 41398_2020_1082_MOESM1_ESM.pdf]

"Synaptic processes and immune-related pathways implicated in Tourette Syndrome" by Tsetsos et al.

Supplemental material

Eigenvector	p-value
Eigenvector 1	3.47946e-08
Eigenvector 2	4.16711e-06
Eigenvector 3	1.2927e-07
Eigenvector 4	2.50729e-07
Eigenvector 5	1.22125e-15

Supplementary Table 1: Significant eigenvectors according to the Tracy-Widom test employed by EIGENSOFT. Eigenvectors with p-value less than 4.44e-6 (0.05/N-1 where N is the number of eigenvectors produced by PCA) are considered significant. In this analysis, the first five eigenvectors were significant and were used to correct for population structure.

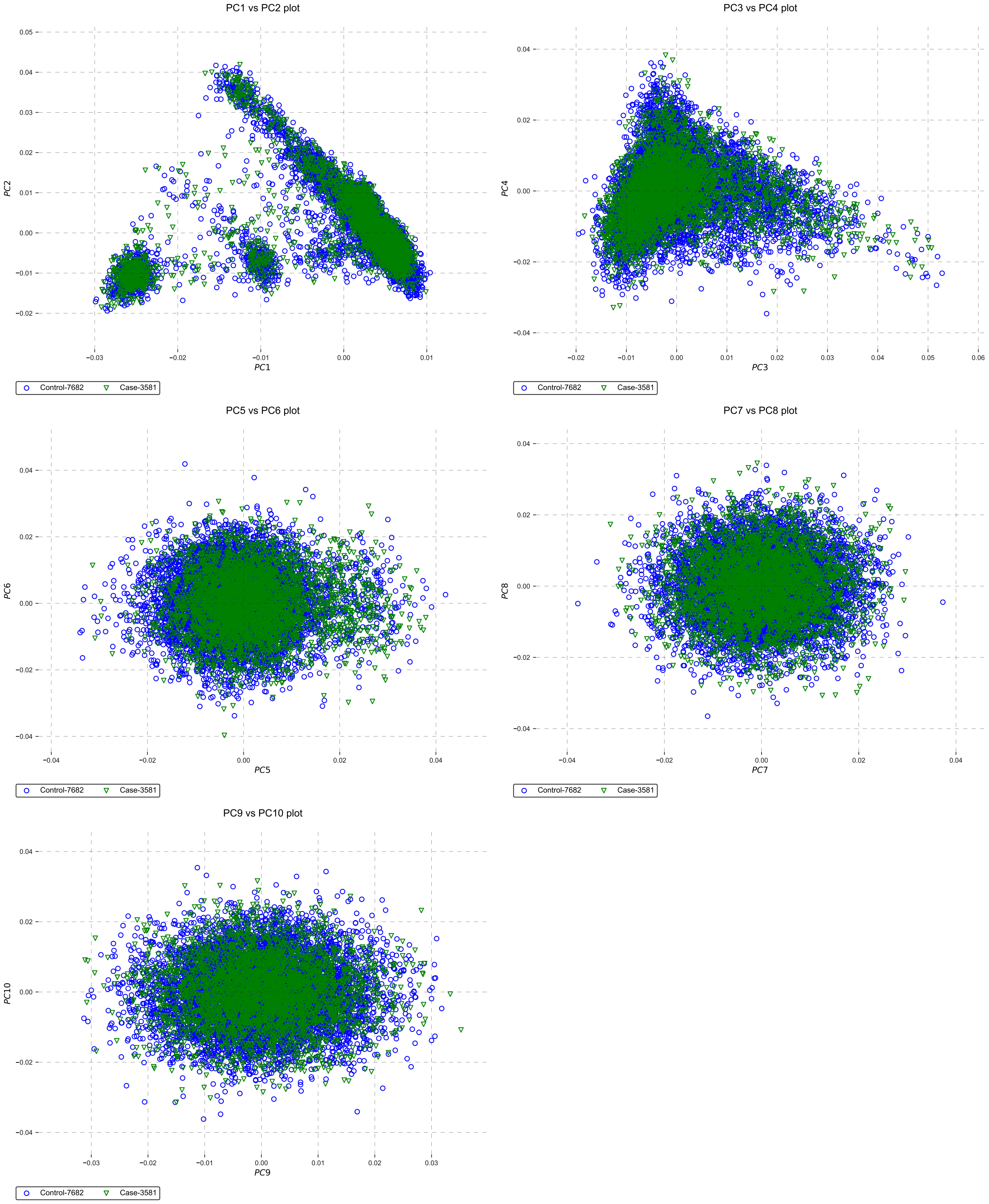

Supplementary Figure 1: Two-dimensional plots depicting the first 10 principal components produced by Principal Component Analysis. We can discern population structure up to the fifth principal component.
